# Incidence, risk factors and mortality of nosocomial pneumonia in Intensive Care Units: A prospective study

**DOI:** 10.1186/1476-0711-3-17

**Published:** 2004-09-15

**Authors:** Emine Alp, Muhammet Güven, Orhan Yıldız, Bilgehan Aygen, Andreas Voss, Mehmet Doganay

**Affiliations:** 1 Clinical Microbiology and Infectious Disease, Faculty of Medicine, Erciyes University, Kayseri, Turkey; 2 Intensive Care Unit, Faculty of Medicine, Erciyes University, Kayseri, Turkey; 3 Medical Microbiology, University Medical Centre St Radboud, Nijmegen, The Netherlands

**Keywords:** Hospital infections, ventilator-associated pneumonia, death rates

## Abstract

To determine the frequency, risk factors and mortality of nosocomial pneumonia a prospective study was conducted in the intensive care units. In the study period, 2402 patients were included. The nosocomial pneumonia was defined according to the Centers for Disease Control Criteria. Overall, 163 (6.8%) of the patients developed nosocomial pneumonia and 75.5% (n = 123) of all patients with nosocomial pneumonia were ventilator-associated pneumonia. 163 patients who were admitted to the intensive care unit during the same period but had no bacteriologic or histologic evidence of pneumonia were used as a control group. The APACHE II score, coma, hypoalbuminemia, mechanical ventilation, tracheotomy, presence of nasogastric tube were found as independent risk factors. Crude and attributable mortality were 65% and 52.6%, respectively. The mortality rate was five times greater in the cases (OR: 5.2; CI 95%: 3.2–8.3). The mean length of stay in the intensive care unit and hospital in the cases were longer than controls (p < 0.0001). Patients requiring mechanical ventilation have a high frequency of nosocomial pneumonia.

## Background

Nosocomial pneumonia (NP) is the most frequent nosocomial infection in the intensive care units (ICU). The reported frequency varies with the definition, the type of hospital or ICU, the population of patients, and the type of rate calculated. In the recent studies, the incidence was reported as 6.8–27% [1-4]. In an one day point prevalence study in European ICUs, ICU-acquired pneumonia accounted for 46.9% of nosocomial infections [5]. The National Nosocomial Infections Surveillance (NNIS) system reported that NP accounts for 31% of all nosocomial infections in intensive care units [6]. The risk of pneumonia is increased in the intubated patients receiving mechanical ventilation (MV) and the ventilator associated pneumonia (VAP) frequencies varied between 7–70% in different studies [7-9]. NP developed at a rate of 0.9 cases per 1000 patient-days in non-ventilated patients versus rates of 20.6 cases per 1000 patient-ventilator-days and 14.8 cases per 1000 patient-days in patients who received any MV [10]. NP is also associated with high morbidity and mortality in ICUs. The increasing incidence of infections caused by antibiotic-resistant pathogens contributes to the seriousness of these infections. The mortality rate reaches to 20–50%, and also NP caused by high-risk pathogens (*Pseudomonas aeruginosa*, *Acinetobacter spp*., *Stenotrophomonas maltophilia*) are associated with higher mortality [1,11,12]. Patients with NP, stay 1 to 2 weeks longer than those without NP and result in higher costs [13].

Studies on NP are mainly reported from the United States and European countries, whereas studies from around the world are missing. The aims of this study were to assess incidence, risk factors and mortality of NP in Eurasian intensive care units.

## Methods

Between February 2001 and February 2002 a prospective study was conducted among intensive care units (ICU) patients of the Erciyes University Hospital. This university hospital is a teaching hospital and full time intensivists care the patients in ICUs. Patients from the surgical ICU (SICU) (24 beds), medical ICU (MICU) (9 beds) and burn unit (7 beds) were included. The SICU consist of 8 neurosurgical (NICU), 8 general surgery (GICU) and 8 cardiac surgery (CICU) beds. Patients older than 16 yr of age were included. The same infection control doctor collected data and intensivist reviewed the diagnosis of pneumonia. Data collection included physical examination findings, APACHE II scores on admission, consciousness, risk factors (intubation, MV, presence of nasogastric tube, enteral nutrition, tracheotomy), prior surgery, immunosuppression, prior antimicrobial and antacid or histamine type 2 (H_2_) blocker therapy, clinical outcome, length of stay in ICU and in the hospital.

163 patients who were admitted to the ICU during the same period but had no bacteriologic or histologic evidence of pneumonia were used as a control group.

In the ICUs infection control doctor collects active surveillance data routinely and empiric antibiotic therapy is directed at the most prevalent and virulent pathogens reported in these data. Appropriate antibiotic therapy included the administration of at least one antibiotic with in vitro activity against the bacterial pathogens isolated from the patient's respiratory secretions, as well as from blood and pleural fluid when applicable [14].

NP was considered when new and persistent (more than 48 h) pulmonary infiltrates not otherwise explained appeared on chest radiographs. Moreover, at least two of the following criteria were also required: 1) fever >38°C; 2) peripheral leukocyte count >10 000/mm^3^; 3) purulent endotracheal secretions with a Gram stain showing one or more types of bacteria [15]. VAP was considered when its onset occurred after 48 h of MV and was judged not to have been incubated before starting MV [16]. Admission APACHE II score was used to determine the severity of the illness, and attributable mortality was registered, as were laboratory values, electrocardiogram, x-ray, and arterial blood gas values.

Extra length of stay was calculated comparing the extra stay after onset of pneumonia in the cases and after a reference date (the mean value of the extra stay after onset of pneumonia in the cases) in the control group.

### Microbiology

Giemsa stains of sputum samples were performed for all patients. Sputum samples, containing more than 25 polimorphonuclear leukocyte (pnl) and less than 10 (×100) epithel were classified as purulent. If necessary, samples were obtained by nasotracheal aspiration. In that case, samples containing more than 10 pnl (×1000) were defined as purulent. Quantitative cultures of all purulent samples were performed using standard methods. Susceptibility testing was performed by disc diffusion method. In the absence of an alternative diagnosis a bronchoalveolar lavage was performed. In some case, pleural fluid was obtained by thoracentesis and examined for cell count, smear, Gram- and Giemsa-staining and microbiological culture.

### Statistical Analysis

All data were evaluated using SPSS. Parameters were compared using univariate and multivariate logistic regression and chi-square tests. Student t test was used to compare the extra length of stay. Data were given as mean ± SD and a p-value of <0.05 was accepted as significant.

## Results

During the study period, 2402 patients were admitted to the ICUs. Distribution of patients by ICU and length of stay in the ICU are shown in table I. Overall, 163 (6.8%) of the 2402 patients developed NP; 105 (5.8%) SICU- and 58 (11.7%) MICU-patients. The demographics of the NP patients and control group are shown in table II. The percentage of NP in NICU, GICU and CICU were 7.8%, 6.3% and 1.2%, respectively. During the study period no burn unit patient developed NP. The incidence of NP in MICU-patients was much higher than in SICU-patients (X^2 ^= 19.7, p < 0.0001). Length of stay in the MICU was significantly higher than in the SICU, 21.3 ± 21.4 versus 16.2 ± 8.8 days, respectively (p < 0.05). Characteristics of patients who developed NP are shown in table III.

**Table I:** Numbers of patients and length of stay in ICU

**ICU**	**No. of beds**	**Total patient (n)**	**Total length of stay (d)**	**Mean length of stay (d)**
**SICU**	**24**	**1806**	**5594**	**3.1**
NICU	8	767	2213	2.9
GICU	8	636	2132	3.4
CICU	8	403	1249	3.1
**MICU**	**9**	**495**	**2086**	**4.2**
**Burn Unit**	**7**	**111**	**1765**	**15.9**
**Total**	**40**	**2402**	**9445**	**3.9**

**Table II:** Demographic Factors of Study Patients*

	**NP patients (n = 163)**	**Control (n = 163)**	**t**	**p**
**Age**	53.30 ± 16.05	51.50 ± 16.87	0,985	>0,05
**Admission APACHE II**	10.86 ± 3.42	10.18 ± 4.56	1,526	>0,05
**Gender**			0,454	>0,05
**Male**	98 (60)	102 (63)		
**Female**	65 (40)	61 (37)		
**Diabetes mellitus**	22 (14)	32 (20)	1,49	>0,05
**COPD**	36 (22)	11 (7)	4,027	<0.001
**Cardiovascular disease**	31 (19)	19 (12)	1,848	>0,05
**Uremia**	36 (22)	12 (7)	3,823	<0.001
**Neoplasia**	29 (18)	17 (10)	1,914	>0,05
**Immunosuppressive therapy**	6 (4)	3 (2)	1,013	>0,05
**Coma**	143 (88)	23 (14)	19,58	<0,001
**Trauma**	35 (22)	85 (52)	6,038	<0,001

* Data presented as mean ± SD or No. (%)

**Table III:** Characteristics of patients

**Characteristics**	**MICU**	**NICU**	**GICU**	**CICU**	**Total**
Age (mean ± SD)	52.9 ± 15.1	50.25 ± 17.57	58.28 ± 14.38	55.00 ± 14.68	53.3 ± 16.1
Admission APACHE II, (mean ± SD)	11.2 ± 3.3	10.43 ± 3.12	11.03 ± 3.67	11.20 ± 6.38	10.9 ± 3.4
NP APACHEII, (mean ± SD)	16.2 ± 5.1	14.73 ± 3.94	16.30 ± 4.79	14.80 ± 6.37	15.6 ± 4.7
Length of stay in ICU (d, mean ± SD)	21.3 ± 21.4	14.42 ± 8.87	19.47 ± 8.20	12.20 ± 3.96	18.0 ± 14.7
Length of hosp. stay (d, mean ± SD)	25.0 ± 22.5	21.60 ± 11.93	24.78 ± 12.13	18.40 ± 8.26	23.5 ± 16.4

Overall, 17% of all patients requiring MV (n-724) developed VAP. Thereby, VAP accounts for 75.5% (n = 123) of all patients with NP (n = 163) during the study period (OR: 5,4; 95% CI: 3,36–8,75; p < 0,001) (Table IV). Mechanical ventilation was more frequently used in MICU patients than SICU patients (X^2 ^= 6.6, p < 0.01). Consequently, the incidence of VAP was higher for MICU- than SICU-patients (X^2 ^= 29.2, p < 0.0001). Furthermore, the length of ventilation was higher for patients admitted to MICU (6.3 ± 4.0) than SICU (5.1 ± 3.7), but the difference was not statistically significant.

**Table IV:** Rate of VAP in ICUs

**ICU**	**No. of patients required MV**	**No. of patients with VAP (%)**
SICU	550	72 (13.1)
NICU	141	38 (27.0)
GICU	157	31 (19.7)
CICU	252	3 (1.2)
MICU	163	51 (31.3)
Burn unit	11	0 (0)
**Total**	**724**	**123 (17.0)**

During the study period patients received 3128 ventilation days, with an average duration of 11.3 ± 10.0 days per ventilated patient. The device-related incidence rate for VAP was 39.3/1000 ventilation days. The incidence per 1000 ventilation days was 41.9 in SICU, 36.6 in MICU, 66.0 in NICU, 38.0 in GICU, and 9.1 in CICU patients. The mean onset day of NP after MV was 4.2 ± 3.9 days.

Univariate analysis suggested the following risk factors for the development of NP: the APACHE II score, coma, COPD, uremia, hypoalbuminemia, MV, tracheotomy, enteral feeding, presence of nasogastric tube and previous treatment with broad-spectrum antibiotic (Table V). However, multivariate logistic regression showed that the APACHE II score (OR: 1.23; 95% CI: 1.13–1.33), coma (OR: 2.83; 95% CI: 1.24–6.47), hypoalbuminemia (OR: 2.23; 95% CI: 1.01–4.93), MV (OR: 3.35; 95% CI: 1.71–6.56), tracheotomy (OR: 6.03; 95% CI: 1.36–26.76) and presence of nasogastric tube (OR: 2.68; 95% CI: 1.33–5.41) were significant independent predictive factors for the development of NP.

**Table V:** Results of univariate analysis of potential risk factors for NP

**Risk Factors**	**OR**	**95% Confidence Interval**	**p**
Age	1.0	0.99 – 1.02	ns
APACHE II	1.3	1.22 – 1.38	<0.001
Coma	6.6	3.75 – 11.48	<0.001
Trauma	1.7	0.93 – 2.97	ns
COPD	3.9	1.91 – 8.01	<0.001
Diabetes mellitus	0.6	0.35 – 1.16	ns
Central nervous system disorder	1.1	0.71 – 1.77	ns
Uremia	3.6	1.78–7.14	<0.001
Hypoalbuminemia	3.3	1.99–5.61	<0.001
Mechanical ventilation	5.4	3.36–8.75	<0.001
Tracheotomy	12.5	3.75–41.89	<0.001
Enteral feeding	13.9	6.38–30.13	<0.001
Presence of nasogastric	6.3	3.89–10.18	<0.001
Previous antibiotic treatment	3.3	1.94–5.62	<0.001
Immunosupressive therapy	2.0	0.50–8.29	ns
Antacids or H_2 _antagonist therapy	0.6	0.25–1.36	ns
Thoracoabdominal surgery	1.0	0.63–1.69	ns

ns: non-significant

187 pneumonia episodes were observed during the study period, resulting in the isolation of 257 microorganisms. The most commonly isolated pathogens were Gram-negative bacteria (85.6%). Among these pathogens, *A. baumannii *(29.6%), *P. aeruginosa *(20.6%), *Klebsiella pneumoniae *(14.4%) were the most common.

Empiric antibiotic therapy was based on previous surveillance cultures and the Gram stain results. Therapy was adjusted according to the reports of susceptibility testing.

Crude and attributable mortality were 65% and 52.6%, respectively. The mortality in patients without NP was 26.4% (Table VI). The risk of death was five times higher for patients with NP (OR: 5.2; 95% CI: 3.2–8.3; p < 0,001). The mortality rates were high in high risk pathogens (Table VII). The appropriateness of the empiric therapy did not contribute to increased mortality (Table VIII).

**Table VI:** Comparisons of outcomes between NP and control group

	**NP group n (%)**	**Control group n (%)**	**X^2^**	**p**
Mortality	106 (65.0)	43 (26.4)	47.5	<0.0001
Improve	57 (35.0)	120 (73.6)		
Attributable mortality	**52.6%**			

**Table VII:** Mortality rates in high risk pathogens

**Microorganism**	**Mortality/Total (%)**
**Gram negative**	**61/97 (62.9)**
*A. baumannii*	31/42 (73.8)
*P. aeruginosa*	19/28 (67.9)
**Gram positive**	**10/15 (66.7)**
*MRSA*	10/14 (71.4)

**Table VIII:** Appropriateness of empiric therapy and mortality

	**Appropriate n (%)**	**Inappropriate n (%)**	**X^2^**	**p**
**Survive**	43/121 (35.5)	14/42 (33.3)	0.005	>0.05
**Death**	78/121 (64.5)	28/42 (66.7)		

The mean length of stay in the ICU and hospital for the patients with NP were 18.04 ± 14.74 days and 23.49 ± 16.44 days, respectively. The mean length of stay in the ICU and hospital for the control group 3.10 ± 3.03 and 9.64 ± 5.08 days, respectively. This difference was statistically significant (p < 0.0001). The extra stay in the control group was 4.36 ± 3.87 and 17.04 ± 14.17 in the patients (p < 0.001).

## Discussion

The incidence of NP was reported different in different studies, which may be justified by the presence of different populations with variable ages, underlying diseases, and other associated risk factors. Incidence ranges from 6.8 to 27% [1-4] and also in this study it was 6.8%. Development of NP varies according to the different type of ICUs. Craven et al. [17] reported that the rate of pneumonia was higher in MICU but the difference was not significant. In the present study, the rate of NP and VAP was significantly higher in MICU than SICU, possible due to the differences in the proportion of patients that needed MV and the duration of MV.

MV increases the risk of NP by 3- to 10-fold [1,18-23], resulting in an VAP incidence of 7 to 70% [7-9]. Generally, the duration of mechanical ventilation increases the risk of pneumonia. Cook et al. [24] reported that the rate of VAP increased 3% per day in the first week of ventilation, 2% per day in the second week, and 1% per day in the third week. In this study, 75.5% of the cases with NP occurred in ventilated patients. From 724 patients who required MV 123 (17%) developed VAP. Accordingly, patients on MV had a 3-fold higher risk to develop NP than the non-ventilated patients. Consequently, the use of non-invasive MV should be preferred whenever possible, since it has lower rates of nosocomial infections [25-27].

Coma was described as another important risk factor for NP. In these patients, local defense mechanisms of the respiratory airway are altered, allowing microorganisms to better attach to and colonize the mucosal surface. Furthermore, depression of the level of consciousness significantly increases the chance of aspiration, and as a result development of NP [3,28]. In our study, comatose patients had a 2-fold increased risk of NP.

The causative agents of NP differ by the study population and diagnostic techniques but generally Gram-negative bacteria are the most common ones [3,4,28-33]. Colonization of the oropharynx, trachea or stomach with Gram-negative pathogens has been identified as a risk factor for NP [15,31]. Also in our study, the most common pathogens were Gram-negative bacteria. Furthermore, prior antibiotic therapy and COPD, leading to colonization with Gram-negative aerobic pathogens, were reported to be risk factors for the development of NP [11,28,30,34]. In our patient population, univariate analysis suggested that previous antibiotic treatment and COPD increased the risk of pneumonia, but interestingly they were not independent risk factors in multivariate analysis. Furthermore, the presence of a naso-gastric tube was found to be a risk factor in our study population. Naso-gastric tubes impair the function of the gastroesophageal sphincter and increase the risk of maxillary sinusitis, oropharyngeal colonization and reflux, all of which may lead to migration of bacteria [35]. Accurate evaluation of nutritional status and early initiation of enteral feeding is important in ICUs patients and can aid to preserve the gastrointestinal epithelium and prevent bacterial colonization. However, it may also increase the risk of gastric distention, colonization, aspiration, and pneumonia. Though, to reduce the risk of NP, it is important to avoid unnecessary enteral nutrition [30]. In univariate analysis, we found enteral feeding as a risk factor, but in multivariate analysis it was not an independent risk factor. For a long time it was assumed that increased gastric pH levels e.g. after the use of antacids, would allow Gram-negative microorganisms to multiply in the stomach, and consequently lead to an increased rate of NP. Our study results confirm what was reported by George et al. [36], namely that the use antacids or H_2 _antagonists did not increase the risk of NP.

In the literature, tracheotomy is described as a significant risk factor for NP. Bronchial colonization during the procedure and (prolonged) continuation of sedation after the procedure will furthermore increase the occurrence of NP [28], a fact that was also seen in our patients. Patients with tracheotomy had a 7-fold increased risk of NP.

The role of advanced age and high APACHE II scores as risk factors of NP are still under discussion. While Kollef et al. [37] report them as significant risk factors, earlier investigations do not support this [1,30]. In our present study, the APACHE II score was a significant risk factor for the development of NP; suggested that the severity of the general condition of the patient was important. Besides, uremia was found as a risk factor in univariate analysis.

Patients with NP have a significantly higher morbidity and mortality [12,35,38]. Heyland et al. [38] reported the crude mortality rate of VAP 23.7% and an attributable mortality rate 32.3%. However, numerous studies have demonstrated that severe underlying illness predisposes patients in the ICU to the development of pneumonia, and their mortality rates are, as a result, high. Survival in patients with NP primarily by the degree of severity of illness at the time of diagnosis [23,39,40]. On the other hand, this does not exclude the possibility that certain subgroups of patients, such as patients with VAP caused by antibiotic resistant bacteria may have had extra attributable mortality rate [1]. In our study, the crude mortality rates for cases and controls were 65.0% and 26.4%, respectively and the mortality rates were highest in high risk pathogens. The mortality rate was five times greater in cases and attributable mortality of NP was 52.6%. Recent clinical investigations suggest that patients receiving inappropriate initial therapy have a greater mortality rate compared to patients receiving antibiotics to which the isolated bacteria were sensitive. However, in this study there was no statistically difference between the mortality rates of the patients who received appropriate and inappropriate initial therapy.

As a result of the increased morbidity, patients with VAP remain hospitalized for 4–17 days longer than controls [35-38]. This observation was confirmed in the present study. The incidence of NP and VAP in MICU were significantly higher than in SICU patients and consequently the length of stay in the MICU was significantly higher than in the SICU.

In conclusion, NP is a major cause of morbidity and mortality in ICU patients. Especially patients on mechanical ventilation are at high risk. Studies determining the impact of "old" and "new" risk factors of NP should repeatedly be performed in order to effectively guide the implementation of preventive measures methods.
